# Biological properties of copper: Antibacterial, osteogenesis of biomaterials

**DOI:** 10.1016/j.bbrep.2025.102251

**Published:** 2025-09-10

**Authors:** Mohammad Javad Salek Rahimi, Abbas Kianvash, Mohmmad Rezvani, Mohammad Yousef Memar

**Affiliations:** a Department of Materials Engineering, Institute of Mechanical Engineering, University of Tabriz, East Azarbaijan, Tabriz, Iran; b Infectious and Tropical Diseases Research Center, Clinical Research Development Unit of Sina Educational, Research and Treatment Center, Tabriz University of Medical Sciences, Tabriz, Iran

**Keywords:** Antibacterial, Biocompatibility, Cu coating, Osteogenesis, Biomaterials

## Abstract

Copper (Cu) has gained significant attention from the researchers on biomaterials due to its important biological roles in the body. Researchers have found that Cu has biological properties, especially in orthopedic applications as an additive element in alloys or bio-compatible coatings. Additionally, Cu has anti-bacterial, angiogenic, and osteogenic properties. The antibacterial properties of Cu provide an alternative to antibiotics and prevent the development of antibiotic resistance. Furthermore, Cu contributes to bone and blood vessel formation and improves the performance of biological materials. This study examines the potential of Cu-containing implant materials in anti-bacterial and bone and blood vessel regeneration.

## Highlights

• Copper-containing biomaterials exhibit strong antibacterial activity against various bacterial strains.
• Cu ions enhance osteogenesis by stimulating mesenchymal stem cell differentiation into osteoblasts.
• Copper promotes angiogenesis by upregulating VEGF and supporting endothelial cell proliferation.
• Ti–Cu and Mg–Cu alloys show excellent antibacterial properties and biocompatibility in implants.
• Copper-modified scaffolds regulate degradation rates and support bone and vascular regeneration.

## 1 Introduction

The need for implants as bone substitutes is increasing worldwide. Bone defects arise from various causes, including accidents, infections, and bone diseases that require tissue removal. By advances in the field of orthopedic surgeries, there have been significant increases in the number of procedures performed to correct bone defects. Implants made of metal alloys have gained great attention due to their high load-bearing capacity, mechanical resistance, and high fracture toughness [1]. Implants used as a substitute for bones must be biocompatible in the body environment. Implants should have a longer lifespan in the body environment to improve bone formation and also replace joints and other body parts to improve their performance. However, there is a 0.5–2 % chance that these materials may cause health complications and heavy medical expenses after surgery. Infection caused by implant materials is one of the most common complications associated with biological materials [2]. In the case of implants, these infections can lead to the development of infections in prosthetic implant materials. These problems can lead to long-term antibiotic treatments and, as a result, prolong the treatment time and require the removal of implants materials from the body [3,4]. To overcome the problem of infection, systemic antibiotic treatment is usually used, but with the spread of bacterial resistance, the use of these drugs may be ineffective [[5], [6], [7]]. One way to address the problem of bacterial resistance is to create coatings with the addition of antibacterial elements such as Cu and Ag. However, the effect of these ions on cell toxicity is critical. Positively charged metal ions released from implants can bind to negatively charged bacterial cell walls, interact with eukaryotic cell walls, DNA, and proteins present in the cytoplasm and ultimately cause cell necrosis. But it has been shown that metals, especially Ag, have toxic effects on human cells [8]. However, Cu is a micronutrient and essential element for various enzymes and does not accumulate in the human body [9]. Cu ions, due to their inherent antibacterial properties, can also induce the polarization of macrophages toward the M1 phenotype [10]. The antibacterial and osteogenic effects of M1 microenvironments have been well established. Another advantage of Cu over other elements is its functionality under various humidity and temperature conditions, attributed to its two ionic states, Cu^+^ and Cu^2+^ [11]. In contrast, silver (Ag) performs optimally only in moist conditions, and its antibacterial efficacy significantly decreases by approximately 20 % at room temperature and low humidity [12]. Cu is an essential trace element required for human growth and catalyzes essential metabolic processes and plays a role in bone formation [13,14]. Cu ions can play an important role in bone healing by stimulating mesenchymal stem cells and differentiating these cells into osteoblasts [15]. This review aims to overview the potential of Cu-containing implants in antibacterial applications, bone regeneration, and vascularization. This study addresses the fundamental challenges associated with the use of implants in the context of infections and bone regeneration, and presents new perspectives for application in next-generation implants.

## 2 Role of Cu in biological processes

**Fig. 1:**
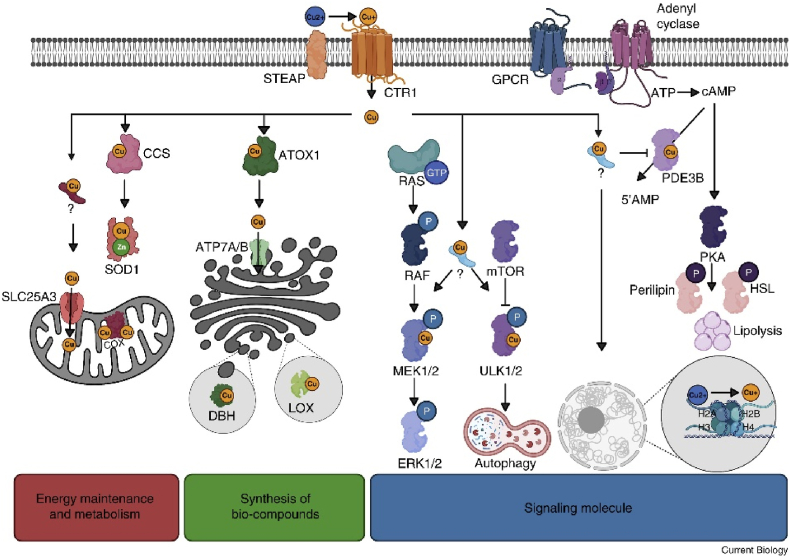
Copper (Cu) as an essential cofactor in enzymatic processes and a dynamic signaling molecule. Cu plays a critical role in various cellular functions, including metabolism, oxidative stress defense, and the synthesis of bioactive compounds. Additionally, Cu regulates key protein kinases and influences cellular processes such as autophagy and cancer progression [19]. (License Number:6080961072603).

Cu is an essential element for normal biological functioning in human's body. This metal is required in small amounts and is vital for the structure and function of many proteins and enzymes. Cu is a rare element that is crucial for regulating organismal function and cannot be synthesized in the body, so it must be obtained through dietary sources. The World Health Organization (WHO) recommends a daily intake of over 2–3 mg of Cu for adults [16]. Cu plays an important role in growth and development of the body, as well as the maturation of nervous, hematological, skeletal, and other systems [17]. Cu is also a critical component of enzymes involved in glucose, amino acid, and cholesterol metabolism and has a unique catalytic role in various reactions [18]. As a transition metal, Cu is biologically converted between different redox states, namely oxidized (II) and reduced (I) Cu. This unique property has made Cu a common catalytic agent for various metabolic reactions in biological systems. It is worth noting that Cu is essential for the healthy functioning of living organisms (Fig. 1).

**Fig. 2:**
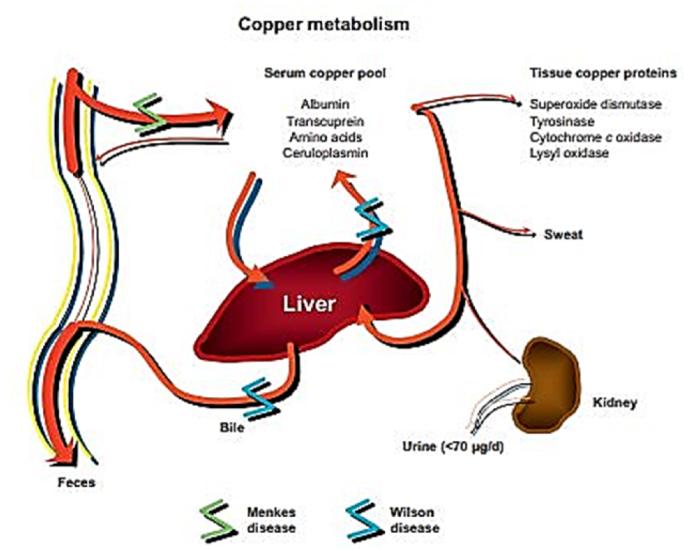
Cu metabolism, Cu deposition in organs, tissues and blood [21]. (License Number:6078761181753).

Furthermore, Cu regulates many cellular processes, including energy metabolism as a central metal ion in cytochrome oxidase and cytochrome *c*, antioxidant activity as part of Cu/zinc superoxide dismutase, dopamine synthesis as a cofactor for dopamine β-hydroxylase, and tissue synthesis as part of Cu-dependent lysyl oxidase in cross-linking collagen in the extracellular matrix and connective tissue [20,21] (Fig. 2).

Unfortunately, Cu and its compounds can be toxic to most organisms and may pose environmental hazards. Therefore, the direct use of Cu and its compounds at higher contents should be limited. However, Cu nanoparticles can serve as an alternative to avoid these consequences.

## 3 Mechanism of Cu as an antibacterial and antiviral agent

The discovery of Cu and its alloys, such as brass and bronze, as natural antibacterial agents with inherent properties to eliminate a wide range of microorganisms has made it possible to use these substances as alternatives to antibiotics and other materials. These alloys have been approved by the US Environmental Protection Agency (EPA) for demonstrating rapid antibacterial activity against *Escherichia coli*, *Staphylococcus aureus*, influenza A virus, adenovirus, and fungi compared to the surfaces of ordinary stainless steel used as handles and touch surfaces [[22], [23], [24], [25]]. Extensive research has been conducted on the use of Cu as an antibacterial agent. Studies have demonstrated the strong antibacterial ability and broad spectrum of SS-type steels containing Cu, such as 304SS and 316L, mainly due to the release of free Cu ions from the surface of the steel. These ions damage the bacterial cell wall and membrane, attract electrons from the bacteria, produce reactive oxygen species (ROS), and consequently cause severe damage and death of bacteria and fungi [26]. Examples of such microorganisms include *Staphylococcus haemolyticus* [27], *E. coli* [28], and *Candida albicans* [29]. During the process of producing ROS, hydroxyl radicals (OH) are generated by photon reactions, often confirmed by the production of ROS such as H_2_O_2_ [[30], [31], [32]]. The hydroxyl radicals produced often damage the cell membrane as a result of the of reactions 2–1 and 2-2:

2-1 2Cu^+^ +2RSH→2Cu^2+^+RSSR+2H^+^

2-2 2Cu^+^ +2H^+^ +O_2_→2Cu^2+^+H_2_O_2_

Additionally, according to the reactions described above, research by Warren's group has shown that the Cu(I)/Cu (II) redox couple and superoxide without OH- play a principal role in the contact killing mechanism in most cases [33]. While many studies have demonstrated that Cu-containing materials have strong antibacterial effects and good biocompatibility, the antibacterial mechanisms associated with Cu^2+^ are still uncertain [34]. Recent research has shown that the presence of lipoprotein carboxyl groups on the surface of bacteria leads to the absorption of Cu ions. When in contact with Cu ions, the permeability of the bacterial cell membrane changes and these cells are damaged. When Cu ions enter bacterial cells, enzyme activity is disrupted, and Deoxyribonucleic Acid (DNA), Ribonucleic Acid (RNA), proteins, and cytoplasm leak out [35].

**Fig. 3:**
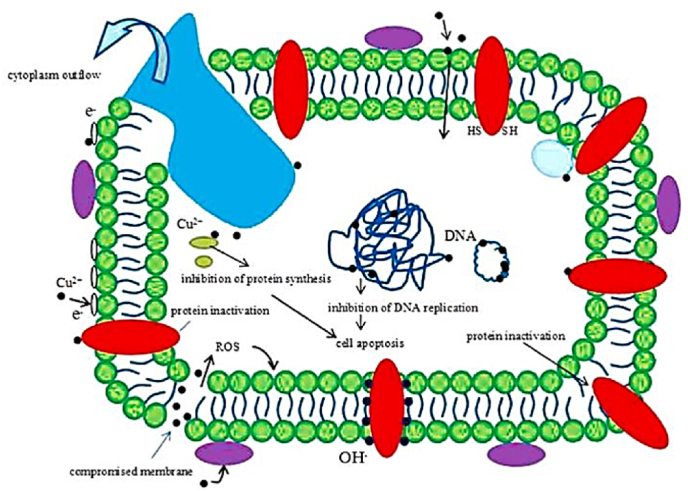
Schematic illustration of the hypothesis about the antibacterial mechanism of Cu^2+^ [36]) Under a Creative Commons Attribution 4.0 International License).

Fig. 3 illustrates the antibacterial mechanisms of Cu, as shown below.

**Fig. 4:**
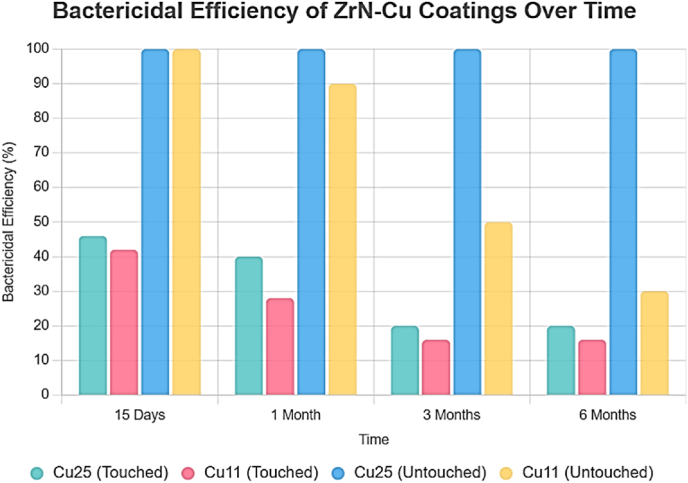
Bactericidal efficiency (%) of ZrN–Cu coatings against *Escherichia coli* (CCM 3988) as a function of exposure time (min). **“Touched”** samples were subjected to repeated mechanical rubbing to simulate real-world contact (e.g., door-handle use), whereas **“untouched”** samples were stored without contact (aging control). Adapted from Behrangi et al. (2024) [40], CC BY 4.0.

Another mechanism by which Cu eliminates bacteria and viruses is through its effect on DNA. In research conducted by Noyce et al., the effect of Cu surfaces was compared to that of stainless-steel surfaces. They found that Cu surfaces can significantly reduce the number of infectious particles of influenza A virus compared to the number present on surface of commonly used stainless steel, through the ability of Cu ions to interfere with DNA by binding to the strands and creating cross-links between them [37]. If similar mechanisms occur with the negative-sense RNA genome of influenza A virus, viral replication can be inhibited by damage to the RNA strands [37]. Recent work has shed light on the mechanical aspects of contact killing, which, along with toxic mechanisms of Cu ions, completely destroy plasmid DNA after cell death by contact killing. The DNA degradation process prevents the transfer of resistance determinants between organisms [38]. The toxicity mechanism of Cu surface in viruses following contact with moist or dry surfaces can vary with contaminated touch surfaces. These results demonstrate the importance of proper surface cleaning protocols to maintain the continued release of Cu ions and prevent the sequestration of ions by pollutants, which can reduce surface efficacy [39]. The research conducted by Behrangi et al. focused on the long-term antibacterial efficacy of ZrN–Cu coatings with varying Cu concentrations of approximately 11 % and 25 % atomic percent. The deposited coatings exhibited 100 % bactericidal efficiency against *E. coli* CCM 3988 within 40 min. In a six-month periodic long-term evaluation, coatings with 25 % atomic Cu demonstrated superior performance. Untouched samples with 25 % Cu showed no reduction in antibacterial efficacy after six months, maintaining 100 % efficiency. In contrast, untouched samples with 11 % Cu began to exhibit reduced efficacy after one month; however, they still achieved 100 % antibacterial efficiency after 160 min of exposure to *E. coli* CCM 3988. Untouched samples with 25 % Cu retained 100 % antibacterial efficacy even after six months of storage, indicating high stability under non-contact conditions (Fig. 4). The primary antibacterial mechanism of ZrN–Cu coatings relies on the release of Cu ions, which disrupt the bacterial cell membrane. However, under contact conditions, the depletion of Cu ions due to the removal of some microparticles from the surface leads to a reduction in antibacterial efficacy. After 15 days of contact, the antibacterial efficiency decreased to 46 % for 25 % Cu and 42 % for 11 % Cu, and after one month, it further declined to 40 % for 25 % Cu and 28 % for 11 % Cu (Fig. 4). The findings of Behrangi et al. suggest that sustained release of Cu ions is critical for achieving long-term antibacterial effects, as these ions are the primary antibacterial agents in Cu-containing coatings [40].

## 4 Mechanisms of bone healing with Cu-containing alloys

**Fig. 5:**
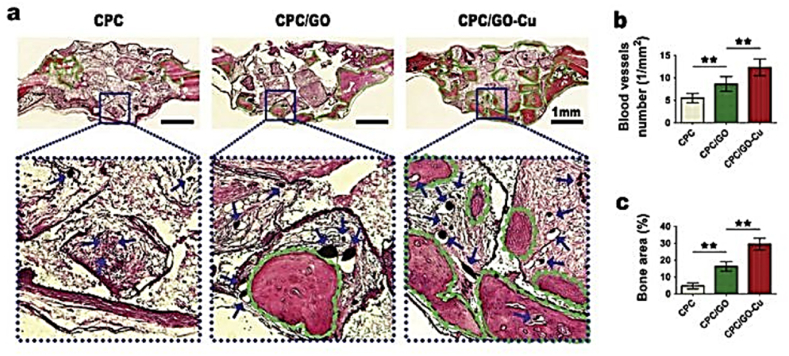
Histological analysis of newly formed bone tissues. (a) Eight weeks after surgery, longitudinal sections were imaged. **Green lines** indicate newly formed bone in the defect areas, and **blue arrows** indicate blood vessels erfused with microfilaments. (b) Quantitative analysis of the number of blood vessels. (c) Quantification of the percentage of newly formed bone surface within defect areas [50]. (License Number: 6078840782641).

Usually, during the wound healing stages, in order to repair damaged tissues, coagulation, hemostasis, inflammation, inhibition and proliferation, which includes the main process of recovery and reconstruction, are performed in 5 stages. Cu ions modulate these phases by upregulating vascular endothelial growth factor (VEGF) during proliferation [42]. Recovery mechanisms are usually complex in which various types of cells and chemical mediators must be activated and coordinated to improve treatment quality. Angiogenesis is the process of forming and growing new blood vessels from pre-existing vessels. This physiological mechanism is essential during the complete wound healing process and plays a vital role in the proliferation phase [41]. Recently, it has been shown that Cu ions bind to and interact with several growth factors involved in blood vessel formation. VEGF is a vital mediator in the angiogenesis process during the proliferative phase. Sun et al. showed that Cu sulfate significantly increased VEGF expression and accelerated wound closure and improved cutaneous wound healing in mice [42]. Phillips et al. have shown that Cu stimulates Matrix Metalloproteinase (MMP) activity at low concentrations and increases MMP expression in fibroblasts at high concentrations [43]. In research by Bernabucci et al. it has been shown that Cu ions can accelerate the growth and maturation of bone collagen enzymes [44]. In the context of tissue, the use of Cu can stimulate the biological properties needed for endothelial cell proliferation during wound healing and increase the differentiation of mesenchymal stem cells into osteoblasts by positively regulating the expression of the VEGF gene [42]. Biocompatibility studies of Ti–Cu alloy show that it has no cytotoxicity to MC3T3-E1 osteoblastic cells [45]. Cu ions enhance the differentiation of mesenchymal stem cells derived from the brain into bone-derived cells, which are related to bone reconstruction [46]. In research works by Wu et al. and Shi et al., the important role of Cu in the context of expressing bone-forming genes such as osteocalcin (OCN), Osteopontin (OPN) and Type I Collagen (collagen I) has been pointed out. [47,48]. Zhang et al. created a scaffold with an oxide-Cu coating and showed that the expression of bone genes (alkaline phosphatase (ALP)) and OCN was higher in the Cu-containing group compared to the control group. The results of Zhang et al.'s study indicated that Cu coatings facilitate cell adhesion and increase growth and one induction capacity in Bone Marrow Stem Cells (BMSCs) (Fig. 5) [49].

**Fig. 6:**
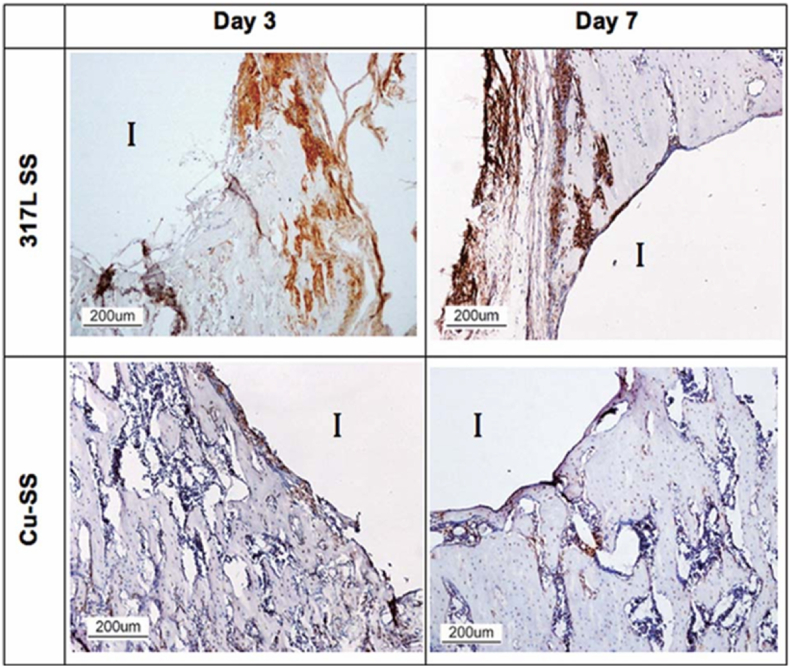
Immunohistochemical staining of TNF- α of bone tissue around the implants after 3- and 7-days implantations. Brown color represents positive staining and ‘I’ the implant location [58]. (License Number 6078850981158).

Zhao et al. have shown that magnesium-Cu-fluorine-containing Ti coatings may promote osteogenesis through ERK 1/2 signaling [51]. Reports indicate that around 18 percent of implant failures are due to aseptic loosening. The phenomenon of aseptic failure is primarily attributed to poor surface osseointegration. Osseointegration is a complex process dependent on both osteogenesis and angiogenesis. Osteogenesis by osteoblasts results in the production of extracellular matrix and proteins. The proteins produced by osteoblasts lead to mineralization. Angiogenesis usually accompanies osteogenesis during bone formation. Bone is a highly vascularized tissue. Blood vessels in bone supply nutrients, oxygen, minerals and growth factors [52]. When the distance between cells and the nearest blood vessel exceeds 100–200 μm, cells die due to lack of nutrients and oxygen. In addition, blood vessels induce osteoprogenitors to sites of cellular damage in bone. Other studies indicate that inadequate vascular supply results in delayed union or nonunion following fracture. Therefore, good angiogenesis may be an effective way to improve bone integration. Cu ions can stimulate the function of endothelial cells to increase angiogenesis [53]. Cu's potential for angiogenesis was initially demonstrated by improving the motility of endothelial cells in cell culture medium containing CuCl_2_. Cu^2+^ released from Cu-containing calcium phosphate cement causes improvement in the growth and expression of angiogenesis-related genes of human umbilical vein endothelial cells (HUVECs). The effect of Cu on bone regenerative scaffolds is also very important. Degradation of bone regenerative scaffolds is crucial for tissue remodeling. The degrading function of scaffolds not only relates to the mechanical properties of the scaffold itself but also to the stability of the repairing environment. Too rapid degradation leads to scaffold instability and makes it difficult to provide sufficient mechanical support while too slow degradation hinders new tissue growth and induces chronic inflammatory response [54]. Research has shown that adding Cu into biomaterials affects their degradation rate. The influence of Cu on the degradation rate varies. The degradation rate of some scaffolds like Cu-containing calcium phosphate increased while others decreased [55]. Komuri observed that after addition of Cu nanoparticles, the biodegradation rate gradually decreased and hence provided better stability for cells [56]. However, the degradation rate is also influenced by the Cu concentration. For example, Lin et al. found that the low Cu dose in scaffolds did not significantly alter the degradation rate but higher Cu concentration increased the degradation rate of the scaffold [57]. In fact, Cu can alter the degradation rate. However, different materials have different effects. The in vivo performance evaluation of Cu-containing coatings indicates their promising success. In studies conducted by Ren et al., the in vivo behavior and multifunctional bioactivity of Cu-bearing 317 stainless steel were investigated in a rat femoral defect model. This research focused on the role of Cu^2+^ ions in osteogenesis and the bone repair process. In Cu-SS alloys, these ions can directly contribute to tissue regeneration by stimulating new bone formation. The functional outcomes of this stimulation are evident in the enhanced bone mineral density (BMD) observed in newly formed tissues, suggesting improved osseointegration between the implant and surrounding bone. Moreover, Cu-SS alloys demonstrated a reduction in inflammatory markers such as TNF-α, leading to excellent biocompatibility and a diminished inflammatory response (Fig. 6). This attenuation of inflammation may significantly contribute to improved bone healing and the long-term success of the implant [58].

**Fig. 7:**
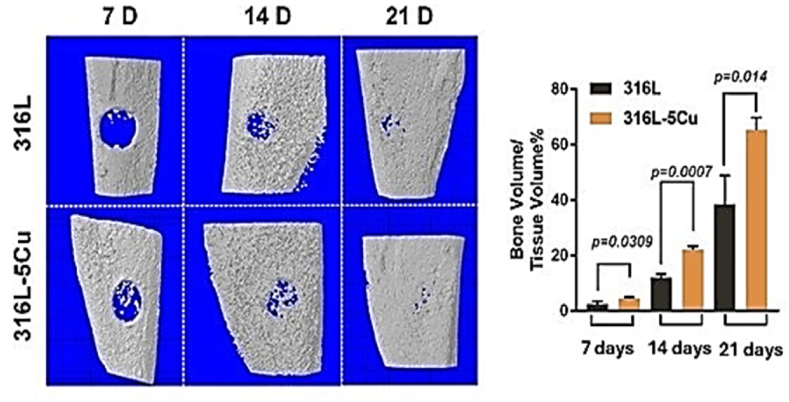
Three-dimensional microcomputed tomography (μCT) imagesofthedrillholesin316L/316L−5Cu-insertedmice7,14, and 21daysafter injury. The ratio of bone volume to tissue volume (BV/TV), representing bone formation in the drill holes, was calculated. Data were statistically analyzed by Student'st-test. Results are presented as mean ± SEM. n = 6 per time point per group [59]. Adapted/Reproduced from Xu D et al., Frontiers in Bioengineering and Biotechnology 2021, under CC BY 4.0 license.

Statistical analyses related to gene expression and various osteogenic markers can reveal the significant and decisive role of Cu-containing alloys in enhancing osteogenesis and modulating immune responses. In this context, the study conducted by Xu et al. focused on data pertaining to several markers, including PDGF-BB, CD206, the formation of type H vessels, and the bone volume to tissue volume ratio (BV/TV). Quantitative values were reported as mean ± standard error of the mean (SEM) using Student's t-test for statistical evaluation. The findings demonstrated a significant increase in PDGF-BB expression in M2a macrophages (CD206^+^), enhanced infiltration of CD206^+^ cells into the injury site, elevated production of mitochondrial reactive oxygen species (mtROS), and accelerated callus formation in the 316L−5Cu group, accompanied by a higher BV/TV ratio compared to the control groups (uncoated 316L alloys). Notably, the BV/TV ratio in the 316L−5Cu group was approximately twice that of the control groups (p < 0.05) (Fig. 7). Time-course data in Xu et al.’s study were collected at 7, 14, and 21 days (n = 6 per group per time point), confirming the progressive osteogenic response of Cu-containing coatings. The statistical analyses presented in this study provide valuable data for comparing osteogenic outcomes between uncoated samples and Cu-containing coatings, thereby contributing significantly to the assessment of Cu's specific role in promoting osteogenesis [59]. A higher BV/TV ratio can indicate enhanced osteogenesis through various mechanisms**.** This increase in BV/TV occurs via a cascade of sequential events. First, Cu promotes the elevation of mitochondrial reactive oxygen species (mtROS) in M2a (CD206^+^) macrophages. The rise in mtROS can upregulate PDGF-BB, which in turn induces the formation of type-H vessels containing endothelial cells. Ultimately, this angiogenic process works in concert with osteogenesis, leading to faster formation of callus (the initial repair tissue) and new bone. In parallel, copper is known to upregulate VEGF, thereby enhancing endothelial proliferation and coupling angiogenesis with osteogenesis [42]. Together, these pathways provide a plausible explanation for the higher BV/TV observed in 316L−5Cu [42,59].

In the study conducted by Zhou et al. biodegradable coronary stents made from Cu–Zn alloys (Zn-0.8Cu) were implanted into porcine coronary arteries for a duration of 24 months. The in vivo evaluation of these alloy-based stents included invasive imaging techniques such as angiography and optical coherence tomography (OCT). The presence of 0.8 % Cu in the zinc alloy contributed to the functional stability of the stent and facilitated favorable vascular remodeling. Angiographic data revealed a continuous increase in luminal diameter at the stent implantation site over time, with a significant reduction in restenosis rate from 32.9 % at 1 month to 12.0 % at 24 months. This indicates sustained vessel patency and the absence of significant long-term narrowing. Furthermore, OCT confirmed complete endothelial coverage of the stent without any evidence of thrombosis or hyperplastic neo-intimal formation. These clinical findings highlight the potential of Cu in enhancing endothelialization and preventing inflammatory responses, thereby supporting the long-term biostability of the stent and minimizing adverse biological reactions [60].

## 5 Cu-containing biomaterials: synthesis and applications

### 5.1 Ti–Cu alloys

**Fig. 8:**
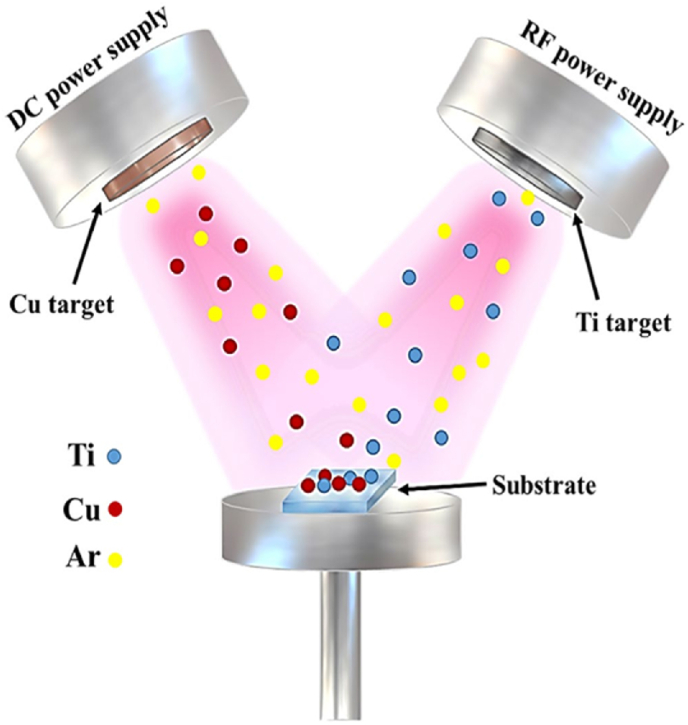
Schematic illustration of the synthesis and preparation of Ti–Cu thin films using the co-sputtering method [65]. (Under a Creative Commons Attribution 4.0 International License).

Titanium alloys containing Cu are relatively safe. Recently, due to their antibacterial activity, Ti–Cu alloys have gained more attention. The most important properties of these alloys include the fluoride hydrogen (HF) etch + anodized Ti–Cu alloy, which has strong antibacterial properties, good biocompatibility, and the ability to promote bone growth. Increasing the Cu concentration in these alloys significantly enhances their antibacterial and bone-forming properties, and a higher and more stable antibacterial rate is achieved with a Cu content of ≥5 % [61]. However, the antibacterial properties of Ti–Cu alloys do not always have a positive correlation with Cu concentration. Zhang et al. found that Ti–5Cu alloy showed a much higher antibacterial rate than Ti–10Cu alloy at different Cu concentrations obtained after various treatments, which could be due to the different Cu forms present in the alloy [62,63]. They also noted that the contact sterilization caused by the Ti_2_Cu phase is the main controlling mechanism of the antibacterial properties of Ti–Cu alloys, and that Ti_2_Cu phases with higher surface areas can have better antibacterial ability. Lin et al. explored the role of pH as an internal trigger in fighting bacterial infections using silk fibroin-based coatings embedded with Cu ions (Ti–Cu@SF) on titanium implants. Their findings showed that under mildly acidic conditions mimicking the environment of bacterial infection the Ti–Cu@SF coatings released elevated levels of Cu^2+^ ions. This targeted ion release proved effective in eliminating resilient bacterial strains such as *E. coli* and *S. aureus*, thereby activating an intrinsic antibacterial response. In contrast, under normal physiological conditions, the coatings released significantly lower levels of Cu^2+^, minimizing potential cytotoxicity and protecting healthy cells. This adaptive release behavior underscores the coating's favorable biocompatibility and smart antibacterial functionality [64]. The release of Cu ions in Ti–Cu thin films and the resulting antibacterial activity achieved through redox reactions and the generation of Cu^+^ and Cu^2+^ species can be highly significant. In the study conducted by Mahmoudi Qashqay et al., Ti–Cu coatings were synthesized via magnetron sputtering deposition. A schematic of the apparatus and the synthesis method is illustrated in Fig. 8. To fabricate these coatings, two sputtering targets RF and DC were employed, corresponding to Ti (purity 99.99 %) and Cu (purity 99.99 %), respectively. To investigate the effect of Cu content, the RF power applied to the Cu target was varied from 10 to 90 W, while all other deposition parameters were kept constant. The study focused on evaluating the antibacterial properties of the coatings, which were produced via co-deposition and compositional control of thin films, against two bacterial strains: Gram-negative (*E. coli*) and Gram-positive (*S. aureus*). With increasing Cu concentration, the surface of the films became rougher and more hydrophilic, which enhanced their resistance to bacterial survival demonstrating up to a 5-log reduction in bacterial count compared to uncoated Ti samples. The release of Cu ions and the reduction reaction from Cu^2+^ to Cu ^+^ facilitated stronger interactions with bacterial membranes, leading to cellular damage. Moreover, the Ti–Cu coatings exhibited high cell viability rates when exposed to L929 fibroblast cells, exceeding 70 %, indicating their non-cytotoxic nature [65].

The influence of Ti_2_Cu precipitates on the antibacterial and biological performance of Ti–Cu alloys have been investigated from various aspects. Xin et al. [63] studied the effect of Ti_2_Cu morphology on corrosion resistance and antibacterial activity, demonstrating that layered Ti_2_Cu (L-Ti_2_Cu) exhibits a significantly higher corrosion rate and more rapid Cu ion release than granular Ti_2_Cu (G-Ti_2_Cu), thereby enhancing antibacterial efficacy. This micro-galvanic behavior makes L-Ti_2_Cu more favorable for use in dental implants due to its superior antibacterial properties. In a related study, Xie et al. [66] fabricated a rough Ti_2_Cu-enriched surface on Ti–3Cu alloys through sandblasting and acid etching. Their results showed that the abundance of Ti_2_Cu particles enhanced the antibacterial activity via micro-potential differences and improved osteoblast adhesion owing to favorable micro/nano-scale surface topography. Additionally, the study by Zhang et al. [67] emphasized that the design of specific heat treatment cycles can modulate the deformation and distribution of Cu within Ti-based alloys, which in turn affects the antibacterial performance by influencing the behavior of Ti_2_Cu precipitates.

### 5.2 Ti–6Al–4V alloys

Adding the Cu element to Ti–6Al–4V can have an improving effect on the mechanical and biological properties. Research done on adding Cu to the Ti–6Al–4V alloy indicates that Ti–6Al–4V–5Cu has better wear resistance and hardness compared to Ti–6Al–4V [68]. Similar research by Hu et al. showed better cell viability and corrosion resistance in Ti–6Al–4V–5Cu in comparison to Ti–6Al–4V. At the same time, this Ti–6Al–4V–5Cu alloy exhibited a special antibacterial ability. Hu et al. attributed the antibacterial ability to contact sterilization through abundant Ti_2_Cu precipitation [69]. The effect of increasing Cu concentration in Ti–6Al–4V-xCu alloys in Ren et al. research showed that the alloys Ti–6Al–4V-xCu (x = 1, 3, 5 wt%) exhibit obvious antibacterial properties with good corrosion resistance and cell biocompatibility. The increased antibacterial capability observed in Ren et al. research enables the wide use of these alloys as implant materials in medical clinical applications [70]. Using suitable heat treatment processes can cause formation of Ti_2_Cu with different concentration, amount and size. In Xu 's research, heat treatment caused increased concentration, amount and size of Ti_2_Cu and increased antibacterial ability through increasing effective chemical contact surface [71].

### 5.3 Mg alloys

Magnesium alloys are widely used in medicine due to their lightweight and load-bearing properties. Adding Cu to these alloys can improve their biological properties, such as resistance to bacteria and stimulation of osteoblasts and endothelial cells. In a study conducted by Liu et al. biodegradable Mg–Cu alloys were designed and fabricated. The addition of Cu to Mg–Cu alloys can increase their mechanical properties, accelerate the creation of an alkaline environment, and release appropriate amounts of biologically active magnesium and Cu to create long-lasting antibacterial effects. The Mg–Cu alloy has no cellular toxicity for HUVEC and MC3T3-E1 cells and creates desirable stimulation for bone and vascular regeneration [72]. In a study conducted by Safari et al., biodegradable Mg–Al–Cu alloys with varying Cu concentrations (0, 0.25, 0.5, and 1 wt%) were fabricated using a two-step process involving mechanical alloying and spark plasma sintering (SPS). Initially, Cu and Al powders with different Cu contents (0, 20, 33, and 50 wt%) were mixed using a ball milling apparatus. The homogenized metal powder mixtures were then consolidated via SPS. The results showed that adding 0.25 wt% Cu to Mg–1Al alloy increases its compressive strength and yield strength due to the formation and homogeneous distribution of the secondary Al_2_Cu phase. Mg–1Al-0.25Cu alloy showed a significant reduction in corrosion rate and a significant increase in the proliferation of MG63 cells compared to pure Mg. Additionally, the release of biologically active Cu and magnesium from Mg–1Al-0.25Cu alloy creates stable antibacterial effects [73]. Li et al. produced cast Mg–Cu alloys with different Cu concentrations of 0.05, 0.1, and 0.25 wt% and evaluated their antibacterial activities. The results showed that Mg–Cu alloys exhibit excellent antibacterial activities against various bacterial strains, including *E. coli*, *Staphylococcus epidermidis*, and Methicillin-resistant *S. aureus* (MRSA). They also demonstrated excellent resistance to bacterial adhesion and biofilm formation [74]. In a study by Zhang et al., the Mg-0.2Cu alloy prepared via casting demonstrated a promising balance between antibacterial efficacy and *in vitro* biodegradability for orthopedic applications [75]. In the study conducted by Liu et al., the effect of pH on biological activities particularly antibacterial properties was investigated. Their research demonstrated that the degradation of Mg alloys results in an alkaline environment with elevated pH levels. This high-pH environment enhances antibacterial activity. However, due to the buffering system present in the human body that regulates pH, this degradation-induced antibacterial effect diminishes over time. As a result, the mentioned antibacterial property of magnesium is primarily effective during the initial stages following implantation [76]. Yan et al. investigated the effect of Cu addition to pure magnesium alloys created by casting and extrusion on the corrosion and biological properties of the alloy. The results showed that the difference in the properties of Mg–Cu alloys depends on their different compositions and microstructures obtained through different processing routes. Galvanic corrosion can be significantly reduced by solution treatment and extrusion due to reduction and good distribution of Mg_2_Cu cathodic phases. The cellular toxicity was minimal with rBMSC incubation. The antibacterial evaluation performed on Mg–Cu alloys showed that the survival of *S. aureus* was reduced compared to pure magnesium due to the release of Cu ions [77].

### 5.4 Stainless steels

Cu addition to 304 stainless steels via forging increased maximum tensile strength (UTS) without affecting microstructure. Addition of 2 wt% Cu to this alloy yielded >80 % antibacterial rate corresponding to released Cu, which none toxic in oral environments [78]. Tang applied solution/aging treatments at 0-3.5 wt% Cu in 316L stainless steel. Aging deposited Cu precipitates, increased yield strength (YS)/UTS. Higher Cu concentration/two-stage aging, yielded good antibacterial performance [79]. Lan investigated Cu's effect on 304 SS alloys' resistance to Streptococcus Mutans/Sanguinis biofilm formation. 5.4 wt% Cu containing 304 SS showed excellent antibacterial activity with minimal NIH3T3 cell toxicity/apoptosis [80]. Ren produced 317L-Cu by stabilizing the appropriate Cu in 317L SS, which releases small Cu^2+^ ions [81]. Akhtar coated 316L stainless steel with chitosan-Cu complexes. These coatings showed strong resistance to *E. coli* and *S. aureus* after 3 h without any bacterial growth [82].

### 5.5 Calcium phosphate bio-ceramics

Calcium phosphates are bio-ceramics that often have chemical and structural similarities with the mineral component of bones [83]. Among them, hydroxyapatite (HA), Ca10(PO4)_6_(OH)_2_, and tricalcium phosphate (TCP), Ca_3_(PO4)_2_) are of great interest either in a mixed or separate form, to produce biphasic calcium phosphates (BCPs) [[84], [85], [86], [87]]. BCPs are novel biomaterials that allow for modulation of biological uptake and ion release due to difference in solubility of the two compounds [88]. The effect of Cu addition to calcium phosphate is of particular interest. Sandrine Gomes et al. have thoroughly investigated the mechanism of Cu doping in BCPs and the behavior of their ion release to determine the cellular toxicity of these bio-ceramics. Their research indicates that higher Cu incorporation is obtained at higher sintering temperatures above 1100 °C, where the poly-morphous phases of α-TCP and Cu_3_(PO4)_2_ decompose to form a Cu-rich HA phase. The presence of mixed Cu^+^/Cu^2+^ among Cu-doped BCP samples identifies several promising bio-ceramics for bone replacement or coating applications. Controlled release of ions from compacted BCP powder to achieve acceptable or excellent cellular toxicity levels is essential [89]. Barralet J.al demonstrated the positive effect of Cu in calcium phosphates on endothelial cells, which are the main mediators of angiogenesis [90]. F.Foroutan and colleagues synthesized Cu^2+^-containing calcium phosphate biocompatible glasses with 0, 2, 4, and 6 mol % Cu^2+^ through precipitation at room temperature. Their research showed that increasing Cu concentration decreased the released phosphorus and calcium, while increasing released Cu. The antibacterial activity against *S. aureus* depends on concentration, increasing higher Cu^2+^ content. Cellular compatibility studies showed increase in Saos-2 osteoblast numbers on surfaces of all glass types for up to 5 days, however, the viability was decreased with higher Cu^2+^ content [91].

## 6 Applications of Cu-containing materials

Cu -based alloys and materials have attracted considerable attention in recent years due to their broad and multifunctional properties, leading to a wide range of applications. Cu is notably utilized in the treatment of bone-related diseases. Owing to its osteogenic potential, Cu can play a significant role in reducing postoperative complications. Furthermore, bone tumors can be effectively eliminated upon exposure to Cu-containing materials. For example, Cu-modified beta-tricalcium phosphate scaffolds incorporating mesoporous silica nanospheres (Cu-MSN-TCP) exhibit photothermal activity that enables the destruction of bone tumors. Cu-containing implants have demonstrated anti-osteomyelitis properties and are employed in the treatment of bone infections due to their antibacterial capabilities. Additionally, Cu promotes chondrogenesis and stimulates cartilage formation in mesenchymal stem cells, making it effective in the treatment of osteoarthritis. Cu ions also play a decisive role in the treatment of osteonecrosis by regulating the expression of hypoxia-inducible factor 1-alpha (HIF-1α) and upregulating VEGF [92]. In the context of dental applications, Cu nanoparticles are highly effective in preventing and treating peri-implantitis, primarily due to their ability to inhibit biofilm formation on implant surfaces, which is crucial in combating bacterial infections. Within the oral cavity, Cu nanoparticles can enhance vascularization and bone regeneration properties that are vital for improving the integration of implants with surrounding bone tissue. Materials such as calcium phosphate containing Cu phosphate nanoparticles, Cu-doped biphasic bio-ceramics, and graphene oxide–Cu nanocomposites have shown promising potential in this field. Moreover, Cu-containing implants may also contribute to the prevention of oral infections [93].

## 7 Conclusion

The present review highlights the necessity of achieving a functional balance between osteogenesis and antimicrobial properties. Among all the Cu-containing alloys studied, Ti–Cu and Mg–Cu alloys exhibit optimal performance in balancing bone regeneration and antibacterial effects. The antimicrobial and osteogenic activities are generally mediated by the release of Cu^2+^ ions. These ions exert antibacterial effects by disrupting bacterial membranes, while also facilitating bone repair by upregulating the expression of VEGF, which promotes endothelial cell proliferation. However, degradation kinetics such as the rapid release of Cu^2+^remain a practical challenge. Nevertheless, there are highly promising approaches to modulate Cu^2+^ release, notably pH-responsive silk fibroin coatings and Cu-doped mesoporous/composite scaffolds. Cu-fibroin coatings suppress Cu^2+^ elution at physiological pH but increase release under mildly acidic, infection-mimicking conditions, whereas Cu-doped mesoporous/composite scaffolds enable controlled, sustained Cu^2+^ elution that supports coupled osteogenesis and angiogenesis. Importantly, comprehensive in vivo studies with standardized release criteria are still required to define safe therapeutic windows and ensure long-term efficacy. Future research should focus on balancing and optimizing the Cu^2+^ release rate to minimize cytotoxicity and enhance clinical applicability, particularly in dental and orthopedic implants.

## CRediT authorship contribution statement

**Mohammad Javad Salek Rahimi:** Writing – original draft, Conceptualization. **Abbas Kianvash:** Writing – review & editing. **Mohmmad Rezvani:** Writing – review & editing. **Mohammad Yousef Memar:** Writing – review & editing.

## Ethical approval

This article does not contain any studies with animals performed by any of the authors.

## Funding

no funding was received.

## Declaration of competing interest

The authors declare that they have no known competing financial interests or personal relationships that could have appeared to influence the work reported in this paper.

## Data Availability

Data will be made available on request.
